# A chromosome-level assembly of the widely used Rockefeller strain of *Aedes aegypti*, the yellow fever mosquito

**DOI:** 10.1093/g3journal/jkac242

**Published:** 2022-09-10

**Authors:** Cera R Fisher, Michael Wilson, Jeffrey G Scott

**Affiliations:** Department of Entomology, Comstock Hall, Cornell University, Ithaca, NY 14853, USA; Center for Cell Analysis & Modeling, University of Connecticut Health Center, Farmington, CT 06030, USA; Department of Entomology, Comstock Hall, Cornell University, Ithaca, NY 14853, USA

**Keywords:** genome assembly, whole-genome sequencing, long-read Nanopore sequencing, disease vector, insecticide susceptible strain, comparative genomics

## Abstract

*Aedes aegypti* is the vector of important human diseases, and genomic resources are crucial in facilitating the study of *A. aegypti* and its ecosystem interactions. Several laboratory-acclimated strains of this mosquito have been established, but the most used strain in toxicology studies is “Rockefeller,” which was originally collected and established in Cuba 130 years ago. A full-length genome assembly of another reference strain, “Liverpool,” was published in 2018 and is the reference genome for the species (AaegL5). However, genetic studies with the Rockefeller strain are complicated by the availability of only the Liverpool strain as the reference genome. Differences between Liverpool and Rockefeller have been known for decades, particularly in the expression of genes relevant to mosquito behavior and vector control (e.g. olfactory). These differences indicate that AaegL5 is likely not fully representative of the Rockefeller genome, presenting potential impediments to research. Here, we present a chromosomal-level assembly and annotation of the Rockefeller genome and a comparative characterization vs the Liverpool genome. Our results set the stage for a pan-genomic approach to understanding evolution and diversity within this important disease vector.

## Introduction

*Aedes aegypti* is a cosmopolitan mosquito species that is an important vector of arthropod-borne viruses (arboviruses), including dengue. Genomic resources are crucial in facilitating the study of *A. aegypti* and its interactions with its pathogens, ecological disturbances, and pesticide applications. However, *A. aegypti* has several genomic characteristics that complicate sequencing and assembly of the full genome. It possesses a relatively large and highly repetitive genome: between 1.2 and 1.4 Gbp long, around 60% interspersed repeats, with only 3 chromosomes (Nene *et al.* 2007; Timoshevskiy *et al.* 2013; Matthews *et al.* 2018). For comparison, malaria-vectoring *Anopheles* mosquito genomes are between 230 and 270 Mbp with <15% repetitive content (Chakraborty *et al.* 2021).

The current *A. aegypti* reference genome (AaegL5) was published in 2018 (Matthews *et al.* 2018). The strain of *A. aegypti* used for the reference genome is LVP AGWG, an inbred line derived from the lab-acclimated strain called “Liverpool” (LVP, named for the Liverpool School of Tropical Medicine, UK), and is thought to be derived from mosquitos collected in West Africa in the 1930s (Kuno 2010), although recent data suggest that an African origin for LVP is unlikely (Gloria-Soria *et al.* 2019). In toxicology and pesticide resistance research, however, the most used susceptible strain is “Rockefeller” (ROCK, named for the Rockefeller Institute of Medical Research at Princeton, NJ). ROCK was established as a lab colony well before WWI, likely as early as 1881, from a wild population in Cuba (Kuno 2010). Since its isolation predates the usage of chemical pesticides, ROCK is commonly used as the reference insecticide-susceptible strain in studies of resistance (Gloria-Soria *et al.* 2019). In its most recent report on insecticide resistance diagnostic concentrations, World Health Organization (WHO) partner laboratories used ROCK, or one of 2 other more recently established strains (“Bora Bora” or “New Orleans”), but none used LVP, including the Liverpool School of Tropical Medicine (WHO 2022).

The fact that the only chromosome-level genome assembly is from a different strain poses a challenge for research that seeks to uncover the genomic basis of any phenotype characterized with respect to ROCK, as most insecticide resistance phenotypes are. Comparing the resistant genome to LVP will erroneously surface irrelevant variation relative to LVP that is shared by ROCK and the resistant strain. These difficulties can be mitigated by sequencing ROCK individuals alongside a strain with a phenotype of interest and comparing both against LVP. However, analysis is easier and more straightforward with a reference genome that corresponds to the reference phenotype.

Here, we present a chromosome-length scaffolded assembly of the ROCK genome sequenced from a single adult female mosquito. Our assembly compares favorably with the existing AaegL5 assembly, but indicates widespread structural variation between ROCK and LVP genomes. To demonstrate the utility of the ROCK genome assembly, within-population polymorphisms were assessed from gDNA shotgun sequencing of a pool of ROCK mosquitos.

## Materials and methods

### Mosquito strain validation and sample preparation

ROCK mosquitos were acquired in 2013 from the CDC Dengue Branch in Puerto Rico. Mosquitos have been in continuous culture at Cornell University, reared according to standard procedures (Smith, Kasai, and Scott 2018). The susceptibility of the ROCK strain to insecticides has been validated periodically by our group (Smith, Kasai, and Scott 2018; Smith, Tyagi, *et al.* 2018; Smith *et al.* 2019, 2021; Silva *et al.* 2021; Sun *et al.* 2021) using both insecticide bioassays and genotyping to confirm the lack of known resistance mutations. Strain validation following the methods of Smith, Tyagi, *et al.* (2018) was conducted in 2021 January prior to sending samples for DNA extraction and sequencing. Mosquito samples for high-molecular weight DNA extraction were isolated individually in cryotubes, flash-frozen in an ethanol/dry ice bath and stored at −70°C before being shipped overnight to the University of Connecticut Center for Genome Innovation (CGI).

### Genome libraries preparation and sequencing

The genomic sequence of ROCK was produced using both single-molecule long read sequencing [Oxford Nanopore Technologies (ONT), Oxford, UK] and whole-genome shotgun sequencing (Illumina, San Diego, CA). For single-molecule sequencing, high molecular weight DNA was extracted from a single virgin female mosquito using established methods (Rivero *et al.* 2007). Briefly, a single virgin female mosquito was gently homogenized with a mini-pestle in a 1.5 ml microcentrifuge tube containing 25 μl lysis buffer [400 mM Tris–Cl (pH 8), 60 mM EDTA (pH 8), 150 mM NaCl, 1% SDS] and 1 μl RNase-A (New England Biolabs, Ipswich, MA, USA). An additional 425 μl of lysis buffer was added to the homogenate and it was incubated at 37°C for 30 min. After the RNAse treatment, 5 μl of Proteinase K (Promega, Madison, WI, USA) and 45 μl more lysis buffer were added and the mixture was incubated at 55°C for 2.5 h; 5 more microliters of Proteinase K were added for a final incubation at 55°C for 1 h. DNA was extracted with 1× volume (500 μl) phenol/chloroform/isoamyl alcohol (25:24:1) and the aqueous phase removed to a new tube for a second extraction with 500 μl chloroform/isoamyl alcohol (24:1). DNA was precipitated from the final aqueous phase with 0.1× volume 3M sodium acetate and 2× volume 100% ethanol. DNA was quantified by spectrophotometry (Nanodrop 2000, ThermoFisher, Waltham, MA, USA) and size was assessed by capillary electrophoresis (TapeStation 4400 Genomic DNA ScreenTape, Agilent, Santa Clara, CA, USA). Short DNA fragments were removed using size-selective precipitation (Circulomics SRE-XS, PacBio, Menlo Park, CA). The median fragment length after size selection was ∼50 kb, and because length of reads was paramount for this dataset, the DNA was not sheared before sequencing. The sequencing library was prepared using the Ligation Sequencing Kit from ONT. The library was run on a Flongle flow cell (MinION, ONT) for quality control. The results of the Flongle run were screened with centrifuge (v 1.0.4-beta) (Kim *et al.* 2016) to ensure that the library did not contain high amounts of human or bacterial DNA prior to committing to the full sequencing run (Section 1 in Supplementary File 1). After passing screening, the library was sequenced on a single flow cell of a PromethION (ONT).

DNA for the Illumina shotgun sequencing library was extracted as described above, except 5 female mosquitos (abdomen removed) were pooled. The DNA extraction was quantified fluorometrically (Qubit DNA Assay, ThermoFisher) and purity was assessed based on Nanodrop 260/280 and 260/230 ratios. DNA was run on a 2% native agarose gel as a rough check for intactness. DNA was fragmented enzymatically targeting an insert size of 350 bp and Illumina sequencing adaptors were ligated using the Lotus DNA library kit (now called xGen DNA EZ, IDT DNA, Coralville, IA, USA) and unique-dual-indexed adapters (IDT DNA). The resulting library was checked for fragment distribution by capillary electrophoresis (Agilent FragmentAnalyzer) and then sequenced on 1/4th of a lane of an Illumina NextSeq 2000 (P3 chemistry, 1× 200 bp reads).

### Genome assembly

De novo assembly was performed with Canu v 2.1.1 (Koren *et al.* 2017) using reads longer than 10,000 nucleotides (Sections 2 and 3 in Supplementary File 1). Specific options for adjusting overlapping behavior were used to control run-time and hard-drive storage footprint; exact commands used are detailed in Section 3 in Supplementary File 1. Duplicative contigs were removed using the Purge Haplotigs pipeline (v 1.0) (Roach *et al.* 2018) with the corrected long reads produced by Canu (Section 4 in Supplementary File 1). Following one round of purging, we aligned the trimmed Illumina reads to the primary contigs using Bowtie 2 (v 2.3.5) (Langmead and Salzberg 2012) and used the alignments to polish (error-correct) with Pilon (v 1.24) (Walker *et al.* 2014) (Section 5 in Supplementary File 1). An additional round of purging removed only a small number of contigs, and subsequent analysis indicated an acceptably low amount of duplication. Therefore, the remaining contigs were kept as the primary assembly. Scaffolding was performed using the AaegL5 reference assembly and the RagTag pipeline (v 2.0.1) (Alonge *et al.* 2019), using default parameters (Section 6 in Supplementary File 1). BUSCO (v 5.0) (Simão *et al.* 2015; Seppey *et al.* 2019) analysis, measuring expected completeness of the genome, was performed after each stage of the assembly refinement process, using the Insecta database from OrthoDB release 10 (Kriventseva *et al.* 2019) (Section 7 in Supplementary File 1).

### Annotation of repeat content, protein coding genes, and ncRNA

Annotation of repetitive content was performed using RepeatMasker and custom repeat libraries created based on AaegL5 with RepeatModeler2 (Flynn *et al.* 2020) and RepBase (v26.09, released 2021 September) (Bao *et al.* 2015). Two different repeat libraries were created for different use-cases of annotated interspersed repeats: (1) the full repeat library produced by RepeatModeler and (2) a filtered library, from which unclassified repeat models were removed. Known *A. aegypti* gene proteins were removed from both libraries based on BLAST searches (e-value threshold of 1e−10) against the AaegL5 proteome (retrieved via ftp from VectorBase release 48). The full library was used to annotate nonprotein coding repetitive content in the assembly for comparison against the reference and to use for later filtering of short-read alignments for single-nucleotide polymorphism (SNP) calling. The filtered library was used to create a masked version of the assembly and the reference for use in genome-to-genome alignments (Section 8 in Supplementary File 1).

BRAKER2 (Brůna *et al.* 2021) was used for annotation in etp-mode for the ab initio prediction of gene models (Section 9 in Supplementary File 1). Orthology-based hints were created for the scaffolded assembly with ProtHint (Brůna *et al.* 2020) using Arthropoda OrthoDB release 10 (Kriventseva *et al.* 2019) as the database. RNA-seq hints were provided by aligning ROCK female abdomen RNA libraries (Sun *et al.* 2021) against the scaffolded assembly using HiSat2 (Kim *et al.* 2019). The resulting predicted amino acid sequences were annotated by similarity searching and orthology determination using DIAMOND (Buchfink *et al.* 2015) [searching against AaegL5 proteins and Swiss-Prot (release 2018) (UniProt Consortium 2021)], EggNOG (Cantalapiedra *et al.* 2021), and InterProScan (Jones *et al.* 2014) as implemented in the EnTAP pipeline (Hart *et al.* 2020) (Section 10 in Supplementary File 1). The EnTAP annotations were used as input to gFACs to filter the BRAKER2 predictions to keep only those that could be annotated with a protein hit or a recognizable protein domain (Section 11 in Supplementary File 1). BUSCO completeness scores for the predicted proteome were assessed against ODB10 Insecta. Because our BRAKER models appeared to be incomplete, we also performed similarity searching in the reverse direction, using TBLASTN with AaegL5 proteins as the query and the ROCK genome as the subject, using an e-value cutoff of 1e−25 (Section 12 in Supplementary File 1). Since this procedure indicated that the genes missing from our BRAKER2 annotations were not missing from the genome, we performed direct coordinate remapping using Liftoff (Shumate and Salzberg 2021) with the RefSeq version of AaegL5 gene annotations (accession number GCF_002204515.2) as the reference (Section 13 in Supplementary File 1).

### Comparisons to reference assembly

Large-scale alignment of the ROCK scaffolded assembly against AaegL5 (genome to genome alignment) was performed with MUMmer4 (Marçais *et al.* 2018) using hard-masked assemblies made with the filtered repeat library (∼50% masked). The output was filtered to one-to-one best alignments over 10,000 bp long with >85% sequence identity. Filtered results on the chromosome level scaffolds were visualized with MUMmer. Breakpoints (representing insertions, deletions, inversions, and rearrangements) were reported using the show-diff command from MUMmer4 and the results analyzed for specific kinds of variants (Section 14 in Supplementary File 1).

The mitochondrial genome was analyzed separately, both because it is small enough to be tractable for visual inspection of alignments and because the breakpoints analysis initially indicated unexpected (and unlikely) copy number variants. Aligning with the AaegL5 mitogenome (LVP sub-strain AGWG, GenBank accession number MF194022.1) revealed that, due to the length of our sequencing reads and the circular nature of mtDNA, the ROCK mitochondrial contig was over-assembled, with spurious duplication at either end. Trimming was done manually based on the start and end positions of AaegL5 mt and self-vs-self overlaps. Additional mosquito mitochondrial genomes were downloaded from GenBank to better assess the divergence of ROCK from LVP and other *A. aegypti* populations. The accession numbers for the additional *A. aegypti* mitogenomes are EU352212.1 (*A. aegypti* LVP substrain ib12), MK575474.1 (*A. aegypti* from Brazil), OM214530.1, and OM214532.1 (*A. aegypti* from Melbourne, AU). Two additional accessions were added as outgroups: AY072044.1 (*A. albopictus*) and MK575484.1 (*Ochlerotatus vigilax*). The circular sequences were linearized to all start at the *tRNA-Met* gene. Alignments were done in Geneious Prime (v2022.0.2) (https://www.geneious.com) using MUSCLE (Edgar 2004). A phylogeny was constructed from the alignment using MrBayes (Ronquist and Huelsenbeck 2003) with substitution model HKY85+G (2 chain MCMC parameters 2,000,000 iterations, sampling frequency 2,000, burn-in length 100,000).

To investigate whether there is a difference in mapping success for short-read ROCK-derived genomic sequences, we evaluated alignment rates of our single-end Illumina reads against the ROCK assembly and AaegL5. For global (end-to-end) alignments, we used bowtie2 with the—very-sensitive parameter set, and for local alignments, we used bwa mem (Section 5 in Supplementary File 1).

### Genome-wide analysis of polymorphisms

The Illumina library used for polishing the assembly was also used to quantify and map SNP distribution across the genome assembly. Alignments were performed with bowtie2 using the –very-sensitive option. For the purposes of variant calling, 1 kb regions with aligned read counts of less than 20 (4×) or more than 350 (70×) (considering only reads with mapping quality ≥30) were excluded as outliers (Supplementary Fig. 2 in Supplementary File 2). Variant calling was performed using FreeBayes (v 1.3.4) (Garrison and Marth 2012) and filtered to discard indels with vcftools (v 0.1.16) (Danecek *et al.* 2011). The remaining polymorphisms were counted using bedops (Neph *et al.* 2012) and bedtools (Quinlan and Hall 2010) in sliding windows of 750 kb incremented by 250 kb (see Section 15 in Supplementary File 1 for complete command listing). Despite filtering regions for outlier read coverage, SNP count per window was still slightly correlated to read depth (Pearson’s *r* = 0.2) (Supplementary Fig. 3 in Supplementary File 2), therefore we normalized SNP density by dividing the count of SNPs per window by read depth per window (calculated across the same sliding 750 kb windows) to yield SNPs per bases mapped (“bases mapped” referring here to read depth) (Supplementary Figs. 4–6 in Supplementary File 2). Results were analyzed and visualized with R (v 4.1.1) (R Development Core Team 2020) in RStudio (v 2021.09.0 + 351) (R Team 2020) using the packages dplyr (Wickham *et al.* 2021) and ggplot2 (Wickham 2016). We defined regions of extremely high polymorphism and extremely low polymorphism empirically by examining histograms of normalized and log-transformed SNP density (Supplementary Figs. 7 and 8 in Supplementary File 2) and looking for clear breakpoints. The threshold for high (normalized) SNP density was 500 SNPs per bases mapped, and the threshold for low SNP density was <1 SNPs per bases mapped.

## Results and discussion

### Sequencing and draft assembly results

The draft assembly was highly contiguous due to the high quality and length of the input reads. ONT platform sequencing efforts resulted in 114 Gbp of sequence with an N50 of 26,000 bp, ∼89× coverage of the 1.27 Gbp genome. Only reads of 10,000 nucleotides or longer were used as input to the Canu assembler (∼65× coverage). After the first read overlapping stage, the longest reads comprising 40× coverage were retained for assembly. The assembly process resulted in a draft diploid assembly of 3,860 contigs with an N50 of 1.4 Mbp (Table 1). BUSCO analysis indicated that the assembly was missing only 15 single-copy orthologs (97.8% complete), and that there was a high rate of duplication (56.7%), as expected from a diploid assembly. The first round of haplotig purging resulted in a reduced assembly of 1,153 primary contigs whose total length was much closer to the predicted value at 1.43 Gbp.

**Table 1.** Metrics of the impact of each step of the genome assembly and refinement process on contiguity, size, and redundancy.

	No. of contigs	N50 (Mbp)	Length (Mbp)	BUSCO complete	BUSCO duplicate
Canu contigs	3,860	1.438	2,034	97.8%^a^	56.7%
Purge haplotigs (first)	1,153	2.149	1,431	97.7%	12.2%
Pilon polished contigs	1,153	2.151	1,432	99.1%	12.2%
Purge haplotigs (second)	1,111	2.154	1,393	99.1%	10%
RagTag scaffolding	86	447.9	1,393^b^	99.4%	9.9%

a Percentages of complete and duplicated single-copy orthologs from Insecta ODB10; *n* = 1,367.

b Length of full assembly minus “Ns” inserted to represent gaps between scaffolded contigs.

Illumina sequencing yielded 315,775,353 single-end 200 bp reads, ∼59× coverage. After quality trimming, 310,023,583 reads were used for polishing the primary contigs. Polishing resulted in a slight improvement to the BUSCO completeness score (Table 1), likely due to recovering genes that were lost due to sequencing errors in the original. After polishing, another round of haplotig purging under the same parameters produced only a slight improvement to the duplication rate diagnosed by BUSCO; however, more aggressive parameters resulted in losing complete single-copy orthologs. Therefore, no further deduplication was attempted. The final BUSCO duplication score of 9.9% is higher than expected, and an investigation of the coordinates of the duplicated single-copy orthologs suggests that these could be consistent with recent tandem duplication or with incompletely scaffolded contigs (Supplementary Fig. 1 in Supplementary File 2). The polished, primary contig assembly was 1,111 contigs with an N50 of 2.2 M bp (Table 1).

### Scaffolding based on reference alignment

The scaffolded assembly comprised 3 chromosomes, the complete mitogenome, 4 supercontigs, and 87 unplaced contigs. A second round of scaffolding using only the unplaced contigs found that 73 of the unplaced contigs were able to be placed on the reference, and therefore could represent unpurged haplotigs. Only 14 (of 87) could not be placed anywhere on the LVP genome. We performed a BLAST search of the GenBank nt database with the 14 unplaceable scaffolds to determine if they were composed of contaminating reads. Four were aligned with high confidence to *A. aegypti* sequences, 1 was determined to be the result of bacterial contamination, and 9 others had no hit passing threshold. Visual inspection of aligned Illumina reads for those 9 indicated that they were potentially assembly artifacts. Those 9 and the bacterial contig were discarded. Additionally, 1 short contig was found to be an alternately assembled mitogenome and was removed from the final contig set. The final nonredundant assembly was made from 1,100 contigs on 86 scaffolds (Table 1).

### Genome annotation and classification of repetitive content and genes

The amount of repetitive content annotated in the genome was essentially identical to LVP, as expected (Supplementary Table 1). Based on the unfiltered repeat model, 65.7% and 65.8% of the ROCK and AaegL5 genomes, respectively, were annotated as interspersed repeats (Table 2). Approximately 20% of the *A. aegypti* genome is composed of retroelements including 10.5% LINEs and 8% LTRs; an additional 27% of the genome are DNA transposons. Unclassified repeats (“unknowns”) make up nearly 13% of the *A. aegypti* genome.

**Table 2.** A comparison of the new ROCK nonredundant assembly and the reference AaegL5 nonredundant assembly.

	ROCK nonredundant	AaegL5 nonredundant
Number of scaffolds	86	2,310
N50 (unplaced)	2,164,021 bp	40,105 bp
Median length (unplaced)	404,476bp	29,378 bp
Minimum contig length	16,818 bp^a^	500 bp
Total assembly length	1.39 Gbp	1.28 Gbp
Total length on chromosomes	1.32 Gbp	1.2 Gbp
BUSCO completeness scores (Insecta odb10)	C: 99.4% [S: 89.5%, D: 9.7%], F: 0.1%, M: 0.7%	C: 99.2% [S: 96.0%,D: 3.2%], F: 0.3%, M: 0.5%
Annotated protein-coding genes	14,556)	14,677
Annotated lncRNA genes	3,663	3,680
Total interspersed repeats	65.7%	65.8%

a The smallest contig is the mitochondrial genome.

BRAKER2 ab initio gene annotation produced an initial 63,196 gene models, and a predicted proteome of 65,991 amino acid sequences (including alternative transcripts). In similarity searching vs the AaegL5 proteome, 27,760 of these found hits passing our thresholds (e-value <10^−5^, query coverage >50%, target coverage >20%). These hits were duplicative to an extent: of the 28,353 annotated proteins from AaegL5, only 22,321 are represented in the hits. This indicates 6,032 annotated reference genes missing in our predicted proteome, which is likely the result of known difficulties of ab initio protein prediction, namely erroneous gene merging and missing exons (Drăgan *et al.* 2016; Scalzitti *et al.* 2020). BUSCO results for the BRAKER2-predicted proteome were 95.2% complete (75.3% singletons and 19.9% duplicates), with 2.6% fragmented and 2.2% missing (Insect ODB 10, *n* = 1,367 single-copy orthologs).

Closer analysis of a handful of missing *A. aegypti* genes (chosen for their relevance to insecticide resistance) indicated that BRAKER2’s gene model prediction had failed to identify or misidentified exons, though the genomic sequence for those genes was present in the assembly. Thus, based on the high degree of error we expect in the ab initio annotation set, we do not provide it as a supplement to this paper. To be confident that our assembly is representative of the full repertoire of genes, we performed TBLASTN with LVP proteins (28,353 proteins from 14,677 genes) as the query and the ROCK genome as the subject. This resulted in 27,909 protein hits representing 14,606 genes passing threshold. Given the success rate of the similarity searching, we employed Liftoff (Shumate and Salzberg 2021) to search and remap the coordinates of all genes annotated in AaegL5 (RefSeq accession GCF_002204515.2) to the ROCK assembly scaffolds. Of the 19,623 genes (coding and noncoding) annotated in AaegL5, Liftoff found and mapped 19,347 in ROCK. Of the 276 genes that could not be mapped, 202 are identified as transfer RNA genes. Protein-coding genes comprise the remaining 74; of these, 40 are listed as “uncharacterized protein.” Included in the list of unannotated *A. aegypti* genes are *myo-sex* (LOC110678344) and *Nix* (LOC110678376, also known as *rsd-1-like*), both of which are male-restricted sex determination genes (Matthews *et al.* 2018) and should not be present in our female-derived genome.

The remaining missing genes are not clustered together but are dispersed across the length of the AaegL5 chromosomes. This suggests that the failure to identify these genes is not the result of failure to sequence or assemble a single substantial portion of the genome. Rather, these genes are either truly missing from the ROCK genome, or if present, they are too divergent or too fragmented to detect through our methods.

### Comparison to AaegL5

Our ROCK genome assembly is highly similar to AaegL5 for most parameters (Table 2). Notable exceptions are the number of scaffolds in the nonredundant assembly, the N50, median length, and the length of the chromosomes. The fewer scaffolds in ROCK is apparently due to the higher contiguity of the initial (Canu) assembly, as the minimum contig length for ROCK is 16,818 bp while the minimum for AaegL5 is 500 bp. The total assembly length for ROCK (1.39 Gbp) is more than 100 Mbp longer than AaegL5 (1.28 Gbp), but both assembled sizes are within the range of real genome size estimates from flow cytometry (Nene *et al.* 2007; Matthews *et al.* 2018).

**Fig. 1.**
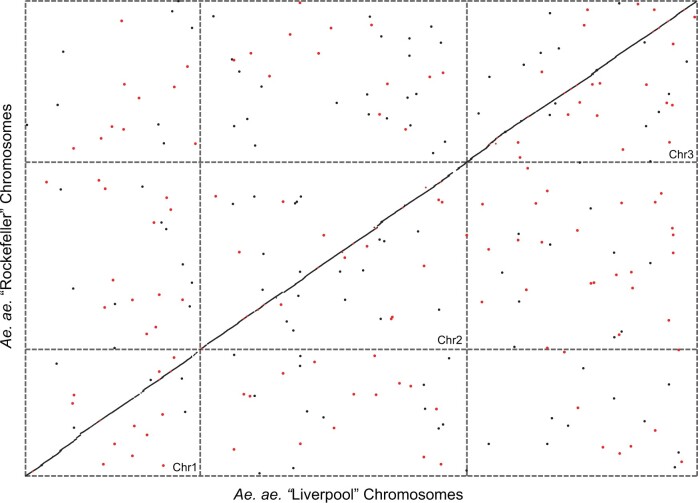
Alignment of chromosome scaffolds of the Rockefeller (ROCK) genome (this study) and the Liverpool genome (AaegL5) shows widespread synteny and agreement between the 2 genomes. This dot plot represents best one-to-one alignments produced by MUMmer with ROCK as subject and AaegL5 as the query, filtered to include alignments longer than 10,000 bp (median = 18,456 bp, longest = 896,729 bp) with minimum % identity = 85%. Note that because even the longest alignment is <0.001% of the length of the genome, each alignment is represented by a dot of the same size. Dots along the diagonal represent the syntenic alignment. Dots off the diagonal represent relocations. Red dots represent reversed alignments.

Alignments visualized with MUMmer indicate that the ROCK genome assembly has large scale synteny with the AaegL5 genome assembly (Fig. 1). However, there are many potential structural variants identified by disjunctions in the filtered alignments (85% identity and 10 kb long or longer) (Table 3). The majority of these are gaps in the alignment (total = 13,220), but a smaller amount are relocations within the same chromosome (573), relocations to a different chromosome (568), and inversions with relocations (905). It is likely the case that a large portion of these SVs are the result of transposable element insertions, given the high proportion of TEs in the genome (Supplementary File 3). These results agree with a similar comparative analysis performed over the much more compact and less repetitive *Drosophila melanogaster* genome, which found that large-scale, gene-impacting structural variation was both more common than anticipated and was often hidden from short-read sequencing (Chakraborty *et al.* 2019).

**Table 3.** The number of breakpoints in the MUMmer (genome-to-genome) alignment for ROCK against AaegL5, broken down by the probable cause of the breakpoint.

	Chr1	Chr2	Chr3	Total
Alignment gap	2,895	5,541	4,784	13,220
Deletion in ROCK	1,375	2,628	2,288	6,291
Insertion in ROCK	1,517	2,910	2,493	6,920
Relocation (same chromosome)	108	254	211	573
Relocation (different chromosome)	118	246	204	568
Duplication in AaegL5	0	0	0	0
Inversion (with possible relocation)	214	349	342	905
Other breakpoint	2	2	2	6

Breakpoints in the alignments are dispersed across the assembly/reference. Alignment gaps may be due to deletion or insertion in ROCK, depending on whether the gap length is positive or negative, respectively. Counts in this table are not an estimate of the number of evolutionary events separating ROCK and LVP, because multiple breakpoints may be seen as the result of a single event; for example, inversions and relocations usually result in 2 breakpoints.

**Fig. 2.**
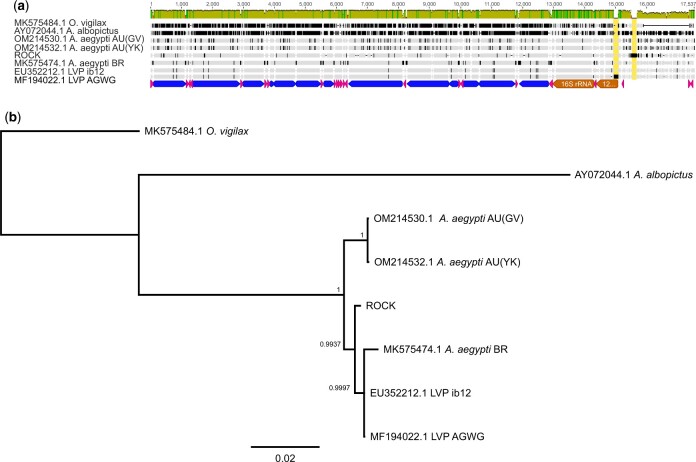
Whole mitochondrial genome phylogeny of *Aedes* mosquitos shows the divergence of the ROCK strain from the Liverpool *Aedes* Genome Working Group (LVP AGWG) strain, in context with other *A. aegypti* accessions and 2 other mosquitos in the *Aedes* genus (sensu lato). a) Alignment of mitochondrial genomes. Control region insertions are highlighted with yellow boxes. Annotations below LVP AGWG show protein coding genes in blue, tRNA genes in pink, and rRNA genes in orange. b) Bayesian phylogeny shows that ROCK groups outside of a clade containing both LVP mitogenomes and a recent accession from Brazil. Numbers on nodes indicate posterior probability. Country of origin abbreviations: BR = Brazil, AU = Australia.

The mitogenome was 16,818 bp long (compared with the LVP length of 16,790 bp). Alignment of the 2 sequences showed a 97.6% pairwise identity, but with 2 large indels in the control region: a 137 bp insertion in LVP (AaegL5 MT: 14,524–14,661), and a 184 bp insertion in the ROCK mitogenome (ROCK MT: 14,832–15,015) (Fig. 2a). The LVP AGWG insertion includes the 5′ end of the 12S ribosomal RNA subunit. Interestingly, neither the LVP nor ROCK insertion was present in the other full mitochondrial sequences available, including the earlier mitogenome accession (EU352212) from the Liverpool ib12 substrain (Nene *et al.* 2007) (an inbred line created for whole genome shotgun sequencing that is a distinct lineage to the LVP AGWG substrain represented by AaegL5). Read mapping strongly supports the 184-bp ROCK insertion. Given that these 2 insertions (in ROCK and LVP) were only found when long-read sequences were used, their presence suggests that other mitogenomes may have similar, as-yet unresolved insertions. Phylogenetic analyses of the mitogenomes indicated that ROCK and LVP were distinct, consistent with what was expected based on their origins (Fig. 2b).

Short-read mapping rates were improved for ROCK-derived Illumina reads against the ROCK assembly relative to AaegL5. For local alignments (more sensitive, but expected to be less specific than global alignments), 99.3% of reads successfully aligned to ROCK vs 98.4% of reads that successfully aligned to AaegL5. The difference was much bigger for global (end-to-end) alignments: 91.3% of reads aligned to ROCK, while only 80.0% aligned to AaegL5.

### Within-population variation across the ROCK genome

**Fig. 3.**
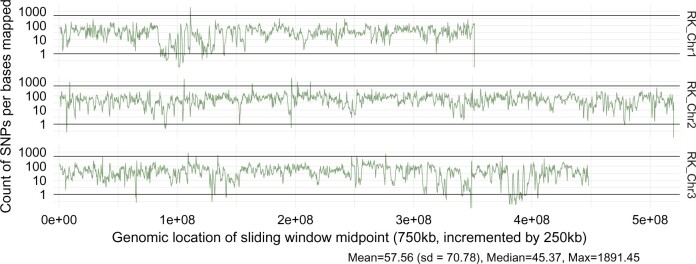
Overview of SNP density and location across the ROCK genome. SNPs were called based on the alignment of a 200-bp Illumina genome library made from pooled DNA of 5 female ROCK mosquitos. Density of SNPs was calculated by counting SNPs in 750,000 bp sliding windows (incremented by 250,000 bp). SNP counts were normalized for differences in read coverage across the genome by dividing by read coverage per window. A log10 *y*-axis scale is used to show both high and low SNP density regions. Black lines at 500 and 1 represent the arbitrary thresholds used to identify regions with high and low SNP density, respectively.

SNPs were found across the ROCK genome, with many notable areas exhibiting signs of depressed polymorphism and a few regions showing increased density of SNPs (Fig. 3). Of the 5,280 sliding windows we evaluated over the genome, 15 had extremely high SNP density (>500 SNPs per bases mapped), with 2 on chromosome 1, 8 on chromosome 2, and 5 on chromosome 3. However, there were many more windows where the density of SNPs was extremely low (<1 SNPs per bases mapped): 39 on chromosome 1, 11 on chromosome 2, and 50 on chromosome 3 (Supplementary Fig. 7 in Supplementary File 2). SNP density is not correlated with the number of genes in each window (Pearson’s *r* = −0.03) (Supplementary Fig. 8 in Supplementary File 2), with the exception that some of the windows with the lowest number of SNPs also have an extremely high count of genes. These regions are good candidates for future characterization of population-level diversity at a finer-resolved scale.

### Conclusions

Herein, we provide the genome sequence for the widely studied ROCK strain of *A. aegypti.* Our assembly provides a valuable resource for the *A. aegypti* community, particularly those engaged in research on vector control strategies, evolutionary biology, and population genetics. Moreover, our approach, combining long-read (but error prone) ONT data with short-read (but accurate) Illumina data, provides a roadmap for low-cost, accurate production and assembly of other strains with research-tractable phenotypes; this assembly cost a little over $5,000, not including salaried labor. This may be particularly necessary for research with organisms whose genomes, like *Aedes aegypti*, are large, contained on few chromosomes, and highly repetitive.

## Supplementary Material

jkac242_Supplementary_File_S1

jkac242_Supplementary_File_S2

jkac242_Supplementary_Table_S1

## Data Availability

All data for this project are available publicly at NCBI under BioProject Accession PRJNA754162. These include raw Nanopore reads passing Q7 threshold (SRA SRR18131520), raw Illumina reads (SRA SRR15429208), and the primary contig assembly (WGS JAKVPO000000000). All computational commands used to produce and analyze this genome are contained in Supplementary File 1. Custom perl and python scripts used for data manipulation tasks, R scripting sessions and session info are publicly available at the github repository for this project, https://github.com/fishercera/Aedes_Rockefeller_Genome. Supplemental material is available at G3 online.
