# Supplementary material for: A chromosome-level assembly of the widely used Rockefeller strain of Aedes aegypti, the yellow fever mosquito
Source: G3 (Bethesda). 2022 Sep 10;12(11):jkac242. doi: 10.1093/g3journal/jkac242 (PMC9635639; doi:10.1093/g3journal/jkac242)
Supplement: jkac242_Supplementary_File_S1 [file jkac242_supplementary_file_s1.pdf]

Supplemental Methods

Software and commands used for assembly and analysis of *Aedes aegypti* "Rockefeller" genome:

Fisher, C. R., M. Wilson, J. G. Scott, 2022. A chromosome-level assembly of the widely used Rockefeller strain of Aedes aegypti, the yellow fever mosquito. *G3 Genes | Genomes | Genetics*.

1) Checking Nanopore output for contamination using Centrifuge

centrifuge/v1.0.4-beta

```
centrifuge -f \
        -x /isg/shared/databases/centrifuge/b+a+v+h/p_compressed+h+v \
        --report-file $READS.report.tsv \
        --quiet \
        --min-hitlen 50 \
        -U $READS
```

2) Filtering out reads shorter than 10kb using

seqkit/v0.10.0
pigz/v2.4)

Reads directly from the PromethION were called by guppy and converted to fastq by the sequencing center. Reads that passed Q7 were then in many small fastq.gz files, each corresponding to a channel on the flow cell. All those files were moved to a single directory, and filtered for reads >10kb and streamed through pigz to compress them into a single file.

```
for i in *.fastq.gz;
do seqkit seq -m 10000  $i | pigz -1 -p 4 >> RK_F3_10kbplus.fastq.gz;
   touch $i.done;
done
```

3) Draft assembly with Canu

canu/v2.1.1
Canu was run on the Xanadu cluster (using SLURM grid manager) at the University of Connecticut's Computational Biology Core (UCONN CBC). A special partition was set up comprising ten nodes each with 40 cores and 192GB RAM. Canu was run with the useGrid mode, with 4 processors and 8GB of RAM provided to the executive job.

Executive command:

```
canu \
-useGrid=TRUE \
-p $LIB\_canu.v1 \
-gridOptions="--partition=general --qos=general" \
-genomeSize=1.3g \
-raw \
-corMhapFilterThreshold=0.0000000002 \
-corMhapOptions="--threshold 0.85 --num-hashes 512 \
--num-min-matches 3 --ordered-sketch-size 1000 --ordered-kmer-size 14 \
--min-olap-length 2000 --repeat-idf-scale 50" \
-mhapBlockSize=500 -ovlMerDistinct=0.975 \
-nanopore $READS
```

The above `-corMhapOptions` are based on the developers' recommendations per the Canu 2.1 usage FAQ at https://canu.readthedocs.io/en/stable/faq.html#my-assembly-is-running-out-of-space-is-too-slow.

Restarts were required at the final 'unitigging' phase because the initial memory allocation by the Canu executive were insufficient.

4) Redundant contig removal with purge_haplotigs

minimap2/v2.15
samtools/v1.7
R/v3.6.0
purge_haplotigs/v1.0

4.1) Read alignment with minimap2 & samtools

```
REF=$1
READS=$2
OUT=$3

minimap2 -t 48 -ax map-ont $REF $READS > $OUT\.sam
samtools view -hF 256 $OUT\.sam \
  | samtools sort -@ 48 -m 2G -o $OUT\.aligned.bam -T $OUT\.tmp.ali

samtools index $OUT\.aligned.bam
```

4.2) Create contig coverage histogram with R, used for picking purging cutoffs

```
purge_haplotigs readhist -b $OUT\.aligned.bam -g $REF
```

4.3) Assign contig coverage groups using cutoffs

The histogram indicated that good cutoffs for ROCK were 5, 35, and 60 (approximately the minima of the curve).

```
LOW=$4    ## 5
MED=$5    ## 35
HIGH=$6   ## 60
purge_haplotigs  contigcov -i  $OUT\.aligned.bam.gencov -l $LOW -m $MED -h $HIGH
```

The output of this stage is coverage_stats.csv

4.4) Purge haplotigs based on coverage_stats.csv

```
mv coverage_stats.csv $OUT\.coverage_stats.csv
purge_haplotigs purge -b  $OUT\.aligned.bam -g ${ref} -c
 $OUT\.coverage_stats.csv -d -a 60
```

5) Error correction with Pilon

bowtie2/v2.3.5.1
samtools/v1.9
pilon/v1.24

5.1) Trim Illumina reads for adapters and quality

Trimmomatic/v0.39

```
wheretrim=/path/to/trimmomatic ## weuse a variable to make the command a little
more legible
lib="library.name" ## a convenient prefix to name each library

java -jar $wheretrim/trimmomatic.jar SE -phred33 -trimlog $lib.trimlog \
      input/scratch/$lib.R1.fastq.gz \
      input/scratch/$lib.R1.trimmed.fastq.gz \
      ILLUMINACLIP:$wheretrim/adapters/Illumina.fa:2:30:10 LEADING:10
TRAILING:10 SLIDINGWINDOW:4:15 MINLEN:60
```

5.2) Align trimmed reads

We made a bowtie2 index from the assembly:

```
FASTA=$1    ## The scaffolded assembly
PRE=$2 ## A short name for the bowtie2 index

bowtie2-build -f $FASTA $PRE
```

Then we aligned the Illumina reads:

```
READS=$1 ## Trimmed, single-end reads
PRE=$2   ## the $PRE from above
LIB=$3   ## A short name for the Illumina library being aligned ("RK_G1")
THREADS=16

echo "Aligning $READS to $PRE with bowtie2"
bowtie2 --seed 3125 --threads $THREADS --very-sensitive \
    --rg-id $LIB --rg SM:$LIB \
    -x $PRE \
    -U <( zcat $READS ) \
    --no-unal --xeq -S $LIB.bowtie2.$PRE.sam

samtools sort -@ $THREADS $LIB.bowtie2.$PRE.sam > $LIB.bowtie2.$PRE.sorted.bam
samtools index $LIB.bowtie2.$PRE.sorted.bam

OUTPUT=$LIB.bowtie2.$PRE.sorted.bam
```

Reads were also aligned with bwa mem for coverage analysis purposes:

```
READS=$1 ## Trimmed, single-end reads
PRE=$2   ## A prefix specifying the bwa index
ID=$3    ## A short ID for the readgroup of the BAM file
LIB=$ID

module load samtools
module load bwa/0.7.17

GEN=ROCK
bwa mem -t 32 -R "@RG\tID:$ID\\tSM:$ID" $PRE $READS | \
samtools view -S -hF 256 -u - | \
samtools sort -T /scratch/$USER - >$ID\_bwa-aln\.$GEN\.bam
date

samtools index $ID\_bwa-aln\.$GEN\.bam
```

5.3) Run Pilon

Pilon was run on the UCHC Xanadu Cluster on a `--partition=himem` node, requesting 16 CPUs and 400 GiB RAM.

```
module load pilon/1.24
echo $PILON
ASM=$1
BAM=$2
PRE=$3

java -Xmx400G -jar $PILON \
    --genome $ASM \
    --unpaired $BAM \
    --output $PRE --fix all \
    --threads 16
```

The output from pilon was run through purge_haplotigs (**Step 4**) a second time.

6) Scaffolding with RagTag

RagTag was installed in a local user directory on the UCHC Xanadu Cluster by cloning the repository from github. (https://github.com/malonge/RagTag). RagTag was run with minimap2 as the aligner, using python3 from anaconda3 (v 4.1.1). The cluster job was allocated 32 CPUs and 32 Gib of RAM.

```
REF=$1 ## Liverpool assembly from VectorBase-48
QUERY=$2 ## ROCK primary contigs ("purged-polished-purged")
PRE=$3 ## A convenient prefix for the output directory ("ROCKv4", because this was the fourth scaffolding attempt after tweaking parameters)

python3 /home/FCAM/cfisher/RagTag/ragtag_scaffold.py --aligner minimap2 --mm2-params "-x asm5 -c" \
    -r -u \
    -s 0.7 \
    -o $PRE -t 32 $REF $QUERY
```

Parameters explanation:

`--mm2-params "-x asm5 -c"` -- This is passed through to minimap2 and tells it to use presets for an assembly with ~5% divergence to the reference, and to output CIGAR notation to the .paf alignment file (so that that file can be converted to SAM if desired).

`-r` -- infer gap sizes where possible (if not set, all gaps are set to 100bp of "N").

`-u` -- Add a suffix to the unplaced contig headers (required for NCBI conformity)

`-s 0.7` -- use a minimum orientation score threshold of 0.7 (goes from 0 to 1)

7) BUSCO completeness assessments

BUSCO/v5.0.0
augustus/v3.3.3

```
DB="insecta_odb10"
ASSEMBLY=$1
LIB=$2
export AUGUSTUS_CONFIG_PATH="/path/to/augustus/3.3.3/config/"

busco -i $ASSEMBLY \
        -o $LIB\_busco -l $DB -m genome
```

Later assessment of the proteome was performed in the same way, except that `-m prot` was used.

8) Building repeat libraries with RepeatModeler and RepBase

8.1) Running RepeatModeler

Dfam TE tools/v1.5
RepeatModeler/v2.0.3
RepBase/v26.09, released Sep 2021

RepeatModeler was run using a Docker image from DFAM consortium (https://github.com/Dfam-consortium/TETools)

Pull Docker image using Singularity

`singularity pull tetools.sif docker://dfam/tetools:1.5`

Make a BLAST database for the Liverpool genome (Vectorbase-48) (using BLAST+ command line interface)

`makeblastdb -in $LVP_FASTA -out LVP -dbtype 'nucl' -title LVP`

Run RepeatModeler using the Liverpool blast database

`singularity exec tetools.sif RepeatModeler -database LVP -pa 24 -LTRStruct -recoverDir $RECOVERDIR`

8.2) Classify repeats library

RepeatModeler's default models are not very informative until after Repeat Classification. To provide good information to the classifier, weused RepBase26.09. we downloaded the .embl.tar.gz file from RepBase, selected the files "invrep.ref cinrep.ref drorep.ref angrep.ref celrep.ref" (Other invertbrates, *Ciona intestinalis*, *Drosophila*, *Anopheles gambiae*, *C. elegans*), concanetated them to a single file (in Embl format), and then converted them to RepeatMasker library format using the `embl2rm.pl` BioPerl script found here: https://bitbucket.org/cornell_bioinformatics/embl2rm/src/master/

`perl embl2rm.pl RepBaseRepeats.embl RepeatsMasker.lib`

`makeblastdb -in RepeatMasker.lib -out RepeatMasker.lib -title RepeatMasker -parse_seqids -dbtype 'nucl'`

Run RepeatClassifier on the repeat model produced from LVP, using the RepBase libraries

`singularity exec tetools.sif RepeatClassifier -consensi LVP_v5_RepeatModeler.consensi.fa -pa 20 -repeatmasker_dir ./RepeatMasker`

Output: `LVP_v5_RepeatModeler.consensi.fa.classified`

8.3) Remove *Aedes aegypti* proteins from the repeat library

This library, LVP_v5_RepeatModeler.consensi.fa.classified, is expected to (and does) include some models that correspond to actual *Aedes aegypti genes*. We don't want to mask those for alignment because they are some of our best information for aligning the genome assemblies.

So we remove them by blastx of the classified consensi models against LVP proteome.

```
makeblastdb -in VectorBase-48_AaegyptiLVP_AGWG_AnnotatedProteins.fasta -out
LVP_prots -parse_seqids -dbtype 'prot'

PROTEINS=$1 # the Liverpool protein blast database
DB=$2 # The classified consensi models database
OUT=$3 # an output file name

blastx \
     -query $DB \
     -db $PROTEINS \
     -out $OUT \
     -evalue 0.00001 \
     -html \
     -max_intron_length 0 \
     -outfmt "6 qseqid sseqid evalue bitscore length qstart qend qlen sstart
send slen qframe sframe" \
     -max_target_seqs 1
```

The models that match will be listed under the qseqid header, which is the first column in the specific tab delimited blast output format.

`cut -f 1 $OUT | sort | uniq > ModelsToRemove.txt`

We filter those out of the consensi using a custom python script available in my utility scripts github repository: [filter_out.py]

The resulting RepeatsLibrary becomes the "full" repeats library. The "filtered" repeats library was created by simply removing all of the filtered repeats that were classified as "unknown":

`grep "Unknown" LVP_v5_RepeatModeler.consensi.fa.classified > UnknownHeaders.txt`

`python filter_out.py UnknownHeaders.txt LVP_v5_RepeatModeler.consensi.fa.classified LVP_v5_RepeatModeler.consensi.filtered.fa`

8.4) Run RepeatMasker using the custom repeat models.

```
LIB=$1 ## LVP_v5_RepeatModeler.consensi.fa.classified (or the filtered one)
FASTA=$2 ## ROCK genome assembly
OUTDIR=$3 ## A convenient output directory name

singularity exec tetools.sif RepeatMasker -pa 24 -q -lib $LIB -xsmall -no_is -
dir $OUTDIR\_soft -gff $FASTA
```

The resulting "soft masked" FASTA file (where repetitive content is lower-cased) can be converted to a "hard masked" file by use of a tiny perl script (softmask-2-hard.pl). Hard-masked FASTAs can be used (if desired) for whole genome to whole genome alignment with MUMmer (below).

9) *ab initio* gene model prediction with BRAKER2

9.1) Create RNA-seq hints from previously published data

sickle/v1.33
hisat2/v2.2.1
samtools/v1.9

These RNA-seq data are available from the NCBI SRA under the bioproject PRJNA753843. For this part of the project, we used the four libraries that were made from ROCK female mosquito abdomens (SRX11720683, SRX11720684, SRX11720687, SRX11720688).

FastQC indicated no adapter content for these reads. RNA-seq reads were trimmed using sickle in single-end mode:

```
READS=$1
LIB=$2

sickle se -f $READS -t sanger -o $LIB.trimmed.fastq.gz -g
```

The trimmed reads were aligned to a HiSat2 database made from the scaffolded genome:

```
# build database
ASM=$1 ## assembly
PRE=$2 ## prefix for the database

hisat2-build -p 8 $ASM $PRE
```

```
# align reads
PRE=$1  ## prefix for the database
READS=$2 ## filename of the RNA-seq reads
LIB=$3   ## Short prefix for the library

hisat2 -x $PRE -U $READS -p 16 -S $LIB\.sam
samtools view -@ 16 -uhS $LIB\.sam | samtools sort -@ 16 -o $LIB\.sorted.bam
samtools index $LIB\.sorted.bam
```

Overall mapping rates were around 97%:

```
bash-4.2$ grep "overall" *stats ## The stdout logs from the run
ROCK1.hisat2run.stats:96.80% overall alignment rate
ROCK2.hisat2run.stats:97.04% overall alignment rate
ROCK3.hisat2run.stats:96.73% overall alignment rate
ROCK4.hisat2run.stats:97.04% overall alignment rate
```

All four libraries' BAM files were then merged into one using samtools merge.

9.2) Create protein hints with ProtHint

ProtHint/v2.6.0

```
/home/FCAM/cfisher/ProtHint/bin/prothint.py --workdir
RK_F3.canu.v4.scaffolds.fasta_ProtHint RK_F3.canu.v4.scaffolds.fasta
../orthodb_proteins.fasta --geneSeeds
RK_F3.canu.v4.scaffolds.fasta_ProtHint/GeneMark_ES/genemark.gtf
ASM=$1 ## RK_F3.canu.v4.scaffolds.fasta
PROTS=$2 ## ../orthodb_proteins.fasta

prothint.py --workdir $ASM\_ProtHint $ASM $PROTS

## the output of this for BRAKER is
RK_F3.canu.v4.scaffolds.fasta.prothint_augustus.gff -> mved to
RK_F3.prothint_augustus.gff
```

9.3) Run BRAKER2

Full details on how to run BRAKER2 and the additional files needed are available at the authors' repository: Gaius-Augustus/BRAKER.

```
ASM=ROCKv4.fasta.softmasked
PROTS=RK_F3.prothint_augustus.gff
BAM=ROCK.merged.bam
OUTDIR=ROCKv4_braker2

braker2 --etpmode \
   --genome=$ASM  \
   --bam=ROCK.merged.bam \
   --hints=$PROTS \
   --useexisting \
   --species=aedes \
   --softmasking \
   --gff3 \
   --cores=24 \
   --workingdir=ROCKv4_braker2
```

10) Orthology determination of BRAKER models with EnTAP

EnTAP/v0.9.0-beta

Full documentation on the usage of EnTAP is available at the authors' website: EnTAP@ReadTheDocs

```
ASM=$1 ## ROCKv4.predicted-proteins.faa
EnTAP --runP  -i $ASM \
    -d diamondDBs/swiss_prot_dmd_v9_9.dmnd \
```

```
    -d diamondDBs/Aegypti_Proteins_dmd_v9_9.dmnd \
    --threads 16 \
    --out-dir $ASM\_out \
    --tcoverage 20 \
    --ontology 0 \
    --ontology 1 \
    --protein pfam \
    --protein cdd \
    --contam bacteria \
    --contam fungi \
    --uninformative uninformative_off.txt \
    --taxon aedes_aegypti
```

11) Alt transcript identification, overlapping exon collapsing, and gene model filtering with gFACs

gFACs/v1.1.2

gFACs is a suite of perl scripts that parses annotation files (gtf/gff), filters gene models, sanitizes/standardizes output, and creates CDS and protein fasta files for the predicted gene set. Full usage information is available at the authors' website: gFACs@ReadTheDocs

gFACs was run on a single desktop computer with 8-core processor and 32G of RAM (gFACs is not multi-threaded). We used gFACs in three passes; first to convert the braker2 gtf to a gene_table, because braker2 gtfs are difficult (time-consuming) to parse. We broke the braker gtf up into eight files, separating GeneMark models and Augustus models for each chromosome (=6) and for the models on unplaced contigs (=2 more), in order to run parsing in parallel. Then we concatenated the resulting gene tables and ran gFACs with the scaffolded assembly provided to produce a predicted proteome multifasta for EnTAP. The final pass was to filter gene models based on EnTAP annotations.

First pass:

```
FASTA=
ENTAP=
GTF=$1
FORMAT=braker_2.1.5_gtf
PREFIX=$1
echo "Getting gene table for $GTF"
## This run -- goes with no filters, no EnTAP, and no fasta. The only goal is to
get a gFACs gene table.
## The gtf file has been edited to remove the file_1_file_1g##### format,
replacing it with just g####
/home/crf92/gFACs/gFACs.pl \
-f $FORMAT -p $PREFIX --no-processing --statistics --distributions exon_position
exon_position_data intron_position intron_position_data -O . $GTF
```

Second pass:

```
FASTA=RK_F3.canu.v4.scaffolds.fasta
ENTAP=
GTF=$1
FORMAT=gFACs_gene_table
PREFIX=$1\_uniq
echo "Getting gene table for $GTF"
echo "Outputs - just getting a gtf and fasta files of cds and proteins."
/home/crf92/gFACs/gFACs.pl \
-f $FORMAT -p $PREFIX --statistics --unique-genes-only --sort-by-chromosome --
fasta $FASTA --get-fasta --get-protein-fasta --create-gtf -O . $GTF
```

Third pass:

```
FASTA=$DDIR/ROCK.v4.scaffolded.fasta
ENTAP=ROCKv4.gFACs.final_annotations_no_contam_lvl4.tsv
GTF=$1
FORMAT=gFACs_gene_table
PREFIX=$2
/home/crf92/gFACs/gFACs.pl \
-f $FORMAT -p $PREFIX --statistics --statistics-at-every-step --create-simple-
gtf --create-gff3 --sort-by-chromosome --entap-annotation $ENTAP --annotated-ss-
genes-only --fasta $FASTA --get-fasta --get-protein-fasta --create-gtf --
distributions exon_position exon_position_data intron_position
intron_position_data -O . $GTF
```

12) Annotation of gene models by BLAST

ncbi-blast/v-2.7.1+

The first step was to make a repeat-masked BLASTdb of the ROCK scaffolds:

```
## convert soft-masked fasta into masking information for BLAST

ASM=$1    ## RK_F3.canu.v4.scaffolds.softmasked.fasta
MASK=$2   ## RK_F3.canu.v4.scaffolds.softmasked.fasta.asnb
TITLE=$3  ## A convenient short name

convert2blastmask -in $ASM -out $MASK -parse_seqids -masking_algorithm repeat -
masking_options "repeatmasker, default" -outfmt maskinfo_asn1_bin

makeblastdb -in $ASM \
    -out $TITLE \
        -parse_seqids \
        -title $TITLE \
        -mask_data $MASK \
        -dbtype 'nucl'
```

12.1) long non-coding RNA (lncRNA) annotation

For identifying putative lncRNA, we wanted to just get the single best hit for each of the annotated ncRNAs from AaegL5.3.

```
A=$1 # non-coding RNA transcripts from LVP
B=$2 # Masked nucleotide blast database
OUT=$3 #Name for the outfile

blastn -db $B -evalue 1e-10 \
    -outfmt "6 qseqid sseqid pident evalue stitle mismatch gapopen qstart qend
sstart send qlen slen" \
    -query $A -max_target_seqs 1 -num_threads 8 -out $OUT.tab
```

12.2) Gene model searching by tBLASTn

Because the BRAKER2 annotations were incomplete, we wanted to make sure we weren't truly missing a large amount of genes that should be present. tBLASTn (amino acid sequence query, translated nucleotide database) was run on the UCHC Xanadu Cluster in an array job to save wall-clock time.

```
THREADS=8
mapfile -t FILE < $FILE_LIST   ## The list of files that the LVP proteins were
split into

CURRENT_FILE=${FILE[$SLURM_ARRAY_TASK_ID]}

echo "This task id is $SLURM_ARRAY_TASK_ID and the file we  want to work on is
${FILE[$SLURM_ARRAY_TASK_ID]}"

PROTEINS=$CURRENT_FILE
DB=$2
OUT=LVP_Peptides_on_ROCKv4_$SLURM_ARRAY_TASK_ID\.tab

echo "The output file will be $OUT"

tblastn \
 -query $PROTEINS \
 -db $DB \
 -out $OUT \
 -evalue 1e-25 \
 -html \
 -num_threads $THREADS \
 -max_intron_length 0 \
 -outfmt "6 qseqid sseqid evalue bitscore length qstart qend qlen sstart send
slen qframe sframe" \
 -max_target_seqs 3 \
```

13) Annotation remapping with Liftoff

Liftoff/v1.6.3 installed via bioconda

Liftoff takes a reference fasta and its annotations, and via minimap2 alignment, maps coordinates of annotations to a target fasta.

```
REFERENCE=/mnt/d/Aedes_Liverpool_Reference/RefSeq/GCF_002204515.2_AaegL5.0_genom
ic.fna
TARGET=/mnt/d/Aedes_Nanopore_Genomes/5_AnnotatedAssemblies/RK/ROCKv4/ROCK.v4.cur
ated.scaffolds.fasta
```

```
ANNOTS=/mnt/d/Aedes_Liverpool_Reference/RefSeq/GCF_002204515.2_AaegL5.0_genomic.
gtf

OUTPUT=ROCKv4.curated.L5-Liftoff.gff3
UNMAPPED=ROCKv4_unmapped_features.txt

CHROMS=/path/to/list/of/chroms.txt


### Call liftoff
/home/crf92/miniconda/bin/liftoff -o $OUTPUT -u $UNMAPPED -g $ANNOTS \
        -p 2 \
        -copies -sc 0.99 \
        -m /home/crf92/minimap2/minimap2 \
        -polish -cds \
        -flank 0.2 -overlap 1.0 \
        $TARGET $REFERENCE

# Effect of non-default parameters:
# -copies -sc 0.99 => looks for multiple copies of genes at a 99% identity
threshold
# -polish => Performs re-alignment of identified exons and adjusts coordinates to
restore coding frame
# -cds => Maybe redundant with -polish; checks cds features to ensure proper
coding frame
# -flank 0.2 => extends alignments past start and stop coordinates of the gene to
flanks 20% of the gene's length; helps find optimal alignments.
# -overlap 1.0 => Allows different gene models to overlap each other by as much
as 100%; necessary for being able to find many of the small non-coding RNA genes.
```

14) Assembly-to-reference alignment and structural variants discovery with MUMmer4

MUMmer/v4.0.2 (manual)

14.1) Alignment with nucmer

These were done with the hard-masked versions of the genomes (filtered/no unknowns, ~50% masked), except that the hardmasked "Ns" in LVP were changed to hardmasked "Xs" to prevent the aligner from considering them. Alignments were run on the UCONN CBC "Xanadu" cluster, requesting 48 cpus and 60 GiB RAM.

```
ASM=$1
REF=$2
OUT=$3

nucmer -c 100 --threads=48 -L 5000 --mum --prefix $OUT $REF $ASM
```

14.2) Filtering

The output of nucmer is a .delta file of all alignments that meet the criteria given. For visualizing the best alignments, we first use `delta-filter`:

```
delta-filter -i 85 -l 10000 -1 $PRE.delta > $PRE.pid85.l10000.1delta
```

These options give the 1-to-1 best alignments in the reference and the query (`-1`) that are longer than 10,000 bp (`-l 10000`) and better than 85% identity (`-i 85`)

14.3) Visualizing

gnuplot/v5.2.2

Then create a dot-plot (synteny plot) with `mummerplot` and gnuplot:

```
DELTA=$1 # the delta file to do mummerplot on
OUT=$2 ## The out prefix
#ASM=$3
#REF=$4
QFILE=$3 # the regions of the query to map
RFILE=$4 # the regions of the reference to map

mummerplot --large --postscript -t $OUT -p $OUT -Q $QFILE -R $RFILE $DELTA
```

For the high-level chromosome-only view, QFILE is the temporary names, the lengths, and the orientations of the ROCK chromosomes:

```
1_RagTag        351562100       +
2_RagTag        520047563       +
3_RagTag        447903560       +
```

and RFILE is the same for LVP:

```
1       310827022       +
2       474425716       +
3       409777670       +
```

14.4) Breakpoints analysis

`show-diff` is used on the unfiltered delta file to report all "breakpoints" in the alignment -- places where synteny is lost.

```
show-diff -r RKv4.on.LVPChroms.delta > RKv4.on.LVPChroms-ShowDiff-Reference.txt
```

The numbers given in **Table 3** are derived from the output of show-diff after parsing and counting in R. The R code used and the session_info() are provided in on github at https://github.com/fishercera/Aedes_Rockefeller_Genome/blob/main/ParseShowDiff.md.

15) Population-level heterogeneity analysis (SNP calling)

The SNP calling pipeline uses bowtie2 alignments that are piped into freebayes to produce a variant call file. The variant call file is then filtered and characterized using bedtools and bedops. This variant calling pipeline was adapted from a tutorial by Noah Reid, UCONN CBC staff

15.1) Align reads

bowtie2/v2.3.5.1
samtools/v1.9

Reads were aligned using the same commands from **step 5.2** above.

15.2) Examine coverage for outliers

BEDtools/v2.9.0
bamtools/v2.5.1
htslib/v1.9

Made a simple BED file of 1000bp windows from the assembly:

```
bedtools makewindows -g RK_F3.canu.v4.scaffolds.genome -w 1000 >
RK_scaffolds.1kb.bed
```

The file `RK_F3.canu.v4.scaffolds.genome` is a two-column text file listing scaffold id and length separated by a tab:

```
1_RagTag        351562100
2_RagTag        520047563
3_RagTag        447903560
MT_RagTag       18375
...
```

Get coverage across those 1kb windows using bamtools and bedtools:

```
bamtools merge -list RK_G1_bamfile.list | \                     ## Originally
written for processing >1 bam file
bamtools filter -in - -mapQuality ">30" -isDuplicate false | \ ## filter by
mapping quality > 30
bedtools bamtobed -i stdin | \                                  ## convert to BED
bedtools map \
-a RK_scaffolds.1kb.bed \
-b stdin \
-c 5 -o mean,median,count \
-g RK_F3.canu.v4.scaffolds.genome | \
bgzip > RK_F3.v4.scaffolds_IlluminaLibraries.coverage_1kb.bed.gz
```

Summarize number of counts per 1kb window (over a region of interest) with htslib and a small awk script:

```
tabix $BED_GZ $REGION | cut -f 6 | sort -g | awk '{x=25}{$1=int($1/x)*x}{print
$1}' | uniq -c
```

15.3) Make targets file that excludes outliers

Based on the output of the `tabix | awk` command above, and after visually examining coverage using R, decided to use only regions with counts between 20 and 350 as targets for freebayes. Column 6 of `RK_F3.v4.scaffolds_IlluminaLibraries.coverage_1kb.bed.gz` holds counts information.

```
# Make targets file:
zcat ROCK_coverage_1kb.bed.gz | awk '$6 > 20 && $6 < 350' | bedtools merge |
bgzip >RK_G1.targets_20-350.bed.gz
tabix -p bed RK_G1.targets_20-350.bed.gz

# Make outliers file:
zcat ROCK_coverage_1kb.bed.gz | awk '$6 < 20 || $6 > 350' | bedtools merge |
bgzip > RK_G1.coverage_outliers_20-350.bed.gz
tabix -p bed RK_G1.coverage_outliers_20-350.bed.gz
```

15.4) Run freebayes, filtering out the outlier regions

nota bene: freebayes is meant to be run on multiple individuals with each individual's reads mapped in a separate experiment.

```
PREFIX=$1 ## A short prefix for the output
OUTLIERWINDOWS=RK_G1.coverage_outliers_20-350.bed.gz
GEN=RK_F3.canu.v4.scaffolds.fasta
OUTDIR=variants_freebayes_wholegenome
mkdir $OUTDIR
bamtools merge -list $OUTDIR/RK_G1.bam.list | \
   bamtools filter -in stdin -mapQuality ">30" | \
   bedtools intersect -v -a stdin -b $OUTLIERWINDOWS -nonamecheck | \
   freebayes -f $GEN --stdin | \
   bgzip -c > $OUTDIR/$PREFIX\-on-ROCK_fb.vcf.gz
```

15.5) Filter VCF file, convert to BED, and count SNPs in specified windows

bedops/v2.4.35
vcftools/v0.1.16
BEDtools/v2.9.0

First filter, keeping only SNPs, requiring a depth of at least 20.

```
REFERENCE_FASTA=RK_F3.canu.v4.scaffolds.fasta
VCF_FILE=$1 ## The gzipped vcf-file freebayes created
LIB=$2      ## A short name for the sequencing library

vcftools --gzvcf $VCF_FILE --indv $LIB --maf 0.1 --min-meanDP 20 --remove-indels
--recode --recode-INFO-all -c |\
   convert2bed -i vcf > $LIB.SNPs.rel2RKv4.bed

## NB: --maf 0.1 (minimum allele frequency) is likely not contributing much to
this filtering, because we are only looking at one sample, and --maf looks at
allele frequency across multi-individual VCF files.
```

Then count SNPs in a sliding window.

```
BED_FILE=$1
LIB=$2
#The file RK_F3.scaffolds is a tab-delimited list of scaffolds and their lengths
in a bed-type format
#1_RagTag        0       351562100
#2_RagTag        0       520047563
#3_RagTag        0       447903560

bedops --chop 750000  --stagger 250000 -x RK_F3.scaffolds.txt | bedmap --echo --
count --sweep-all --delim '\t' - <(cat $BED_FILE) >
$LIB.SNPdensity.Chroms.750kb-250kb.bed
```

The resulting BED file was used for visualizations of SNP density in R. See the R session script and sessionInfo at https://github.com/fishercera/Aedes_Rockefeller_Genome/blob/main/ExploratorySNPDensity.R.
