# Supplementary material for: A chromosome-level assembly of the widely used Rockefeller strain of Aedes aegypti, the yellow fever mosquito
Source: G3 (Bethesda). 2022 Sep 10;12(11):jkac242. doi: 10.1093/g3journal/jkac242 (PMC9635639; doi:10.1093/g3journal/jkac242)

Supplementary Data

1) BUSCO duplication investigation and results

The coordinates of duplicated BUSCOs were extracted from the header files of the translated predicted cds sequences in the BUSCO output directory using bash commands and formatted into a BED-like file using sed, cut, and paste. This is just a way to get the coordinates and the name of the SCO into a BED file, any way that will result in 4-column BED file with the 4th column holding the name of the SCO will do.

```
cd /run_insecta_odb10/busco_sequences/multi_copy_busco_sequences

 grep ">" *.faa | sed "s/[:|-]/\t/g" | sed "s/>//" >
Duplicated_ROCKv4_Headers.txt

 cut -f 2 Duplicated_ROCKv4_Headers.txt > tmp1
 cut -f 3,4 Duplicated_ROCKv4_Headers.txt > tmp2
 cut -f 1 Duplicated_ROCKv4_Headers.txt > tmp3

 paste tmp1 tmp2 tmp 3 > ROCKv4_DuplicatedBuscos.bed
```

Then bedtools was used to sort the bed file.

```
bedtools sort -faidx ROCK.v4.NCBI.version.fasta.fai -i
ROCKv4_DuplicatedBuscos.bed > ROCKv4_DuplicatedBuscos.sorted.bed
```

This bed file was split into two files, each containing the coordinates for one of the duplicated BUSCOs, with a small perl script, posted to the github repository for this project at https://github.com/fishercera/Aedes_Rockefeller_Genome/blob/main/BUSCO_Dupes_splitBeds.pl. The two resulting bed files were used to create chord diagrams in R using the package circlize, in a script available at https://github.com/fishercera/Aedes_Rockefeller_Genome/blob/main/BUSCO_chord_diagrams.R.

The results are suggestive of one of two explanations for the higher-than-expected BUSCO duplication rate: either true tandem duplications, or duplicated contigs that were scaffolded next to each other. A zoom-in on a region (Chr1: 100556000-119190000, 18.6 Mbp) shows the syntenic duplicate BUSCOs.

Supplementary Data Figure S1

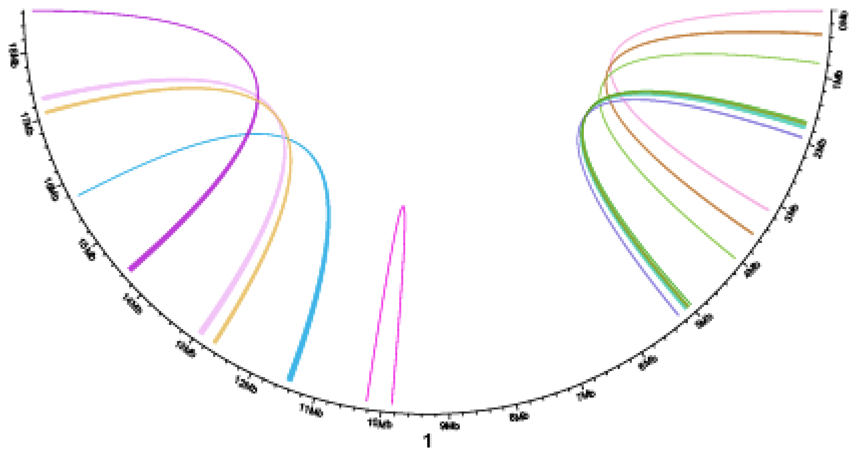

Further investigation is needed to know if these duplicated BUSCOs represent true tandem duplications or regions of the genome that should have assembled into one contig, but for reasons owing to complexity and read heterogeneity, were unable to be overlapped. Attempting to use the purge haplotigs pipeline to further reduce potential redundancy resulted in the loss of complete singleton BUSCOs from our assembly. Rather than simply add those contigs that held BUSCOs back in, and in the interests of creating a genome assembly that captures as much of the full ROCK genome as possible, we have erred on the side of being over-inclusive.

2) ROCK genome assembly coverage by Illumina data

Coverage of Illumina reads was uneven across the genome assembly -- not an uncommon occurrence for highly repetitive genomes.

Mean coverage (read length * read count / span length) for the 1kb windows was 25x, with a very large range. Over the 250kb windows, mean coverage was 21x. Coverage outliers were specified based on the 1kb windows rather than the larger windows, in order to exclude smaller regions/keep more regions with useful data. Plotting the coverage for 1kb windows is RAM-prohibitive, but a small segment gives a good picture of how excluding outliers affected results:

Supplementary Data Figure S2

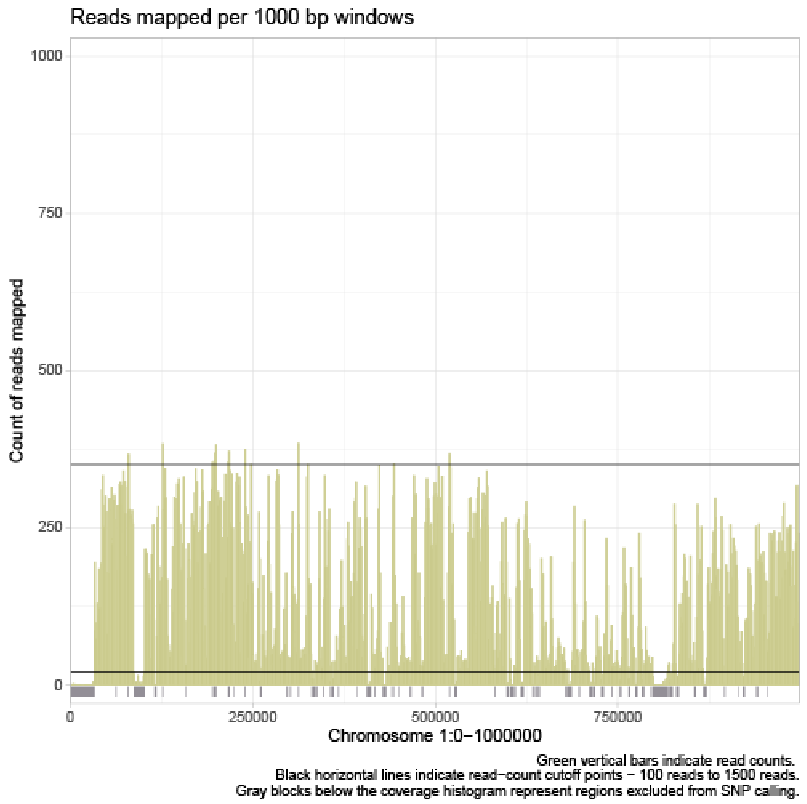

This image shows read coverage in 1kb windows over the first 100,000 basepairs of chromosome 1 (which includes the first five 750kb sliding windows). The lines indicate the cutoffs for "high" (350 reads = 70x coverage) and "low" (20 reads = 5x coverage) coverage outliers, and the grey bands on the bottom depict the width of the windows that were excluded. With small coverage exclusion windows, we are able to keep much of this region where coverage is within tolerance.

3) Coverage effects on SNP counts

Despite excluding coverage outliers, we still saw a mild effect of coverage depth on SNP density.

Supplementary Data Figure S3

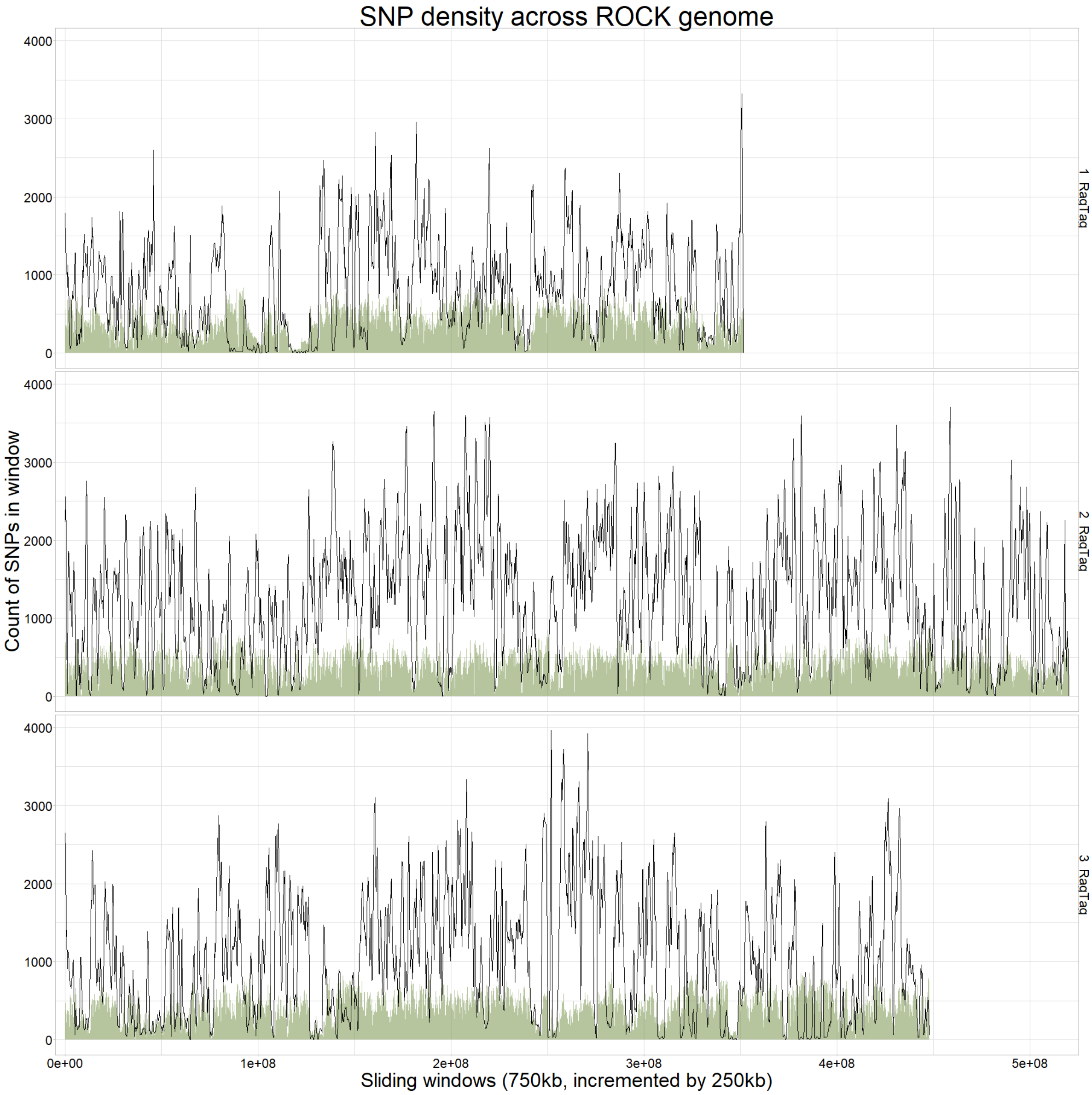

Here, SNP counts per window are represented by the black line, overlaid on a green coverage plot (in 250kb windows). The effect is not strong (Pearson's r = 0.19, a weak positive correlation) but it is obviously apparent particularly in the windows of low SNP counts, as some of them match up clearly with coverage. It is also informative to look at the variance of SNP counts across the genome in histogram plots.

Supplementary Data Figure S4

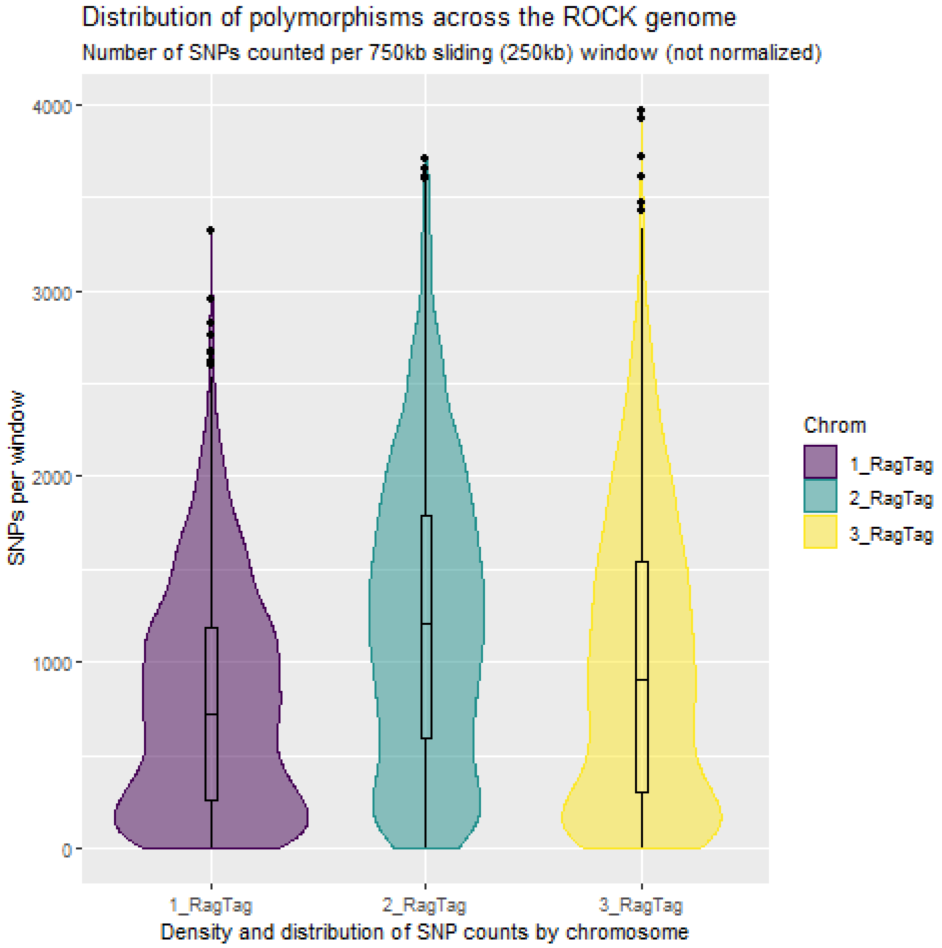

This violin-wrapped boxplot shows the smoothed distribution of windowed SNP counts for each chromosome. Boxes show interquartile range, whiskers show 1.5x interquartile range, with outliers shown as dots. The variance is really high, particularly for chromosome 2!

Supplementary Data Figure S5 Dividing SNP counts by read depth gives us SNPs per bases mapped, which has much lower variance.

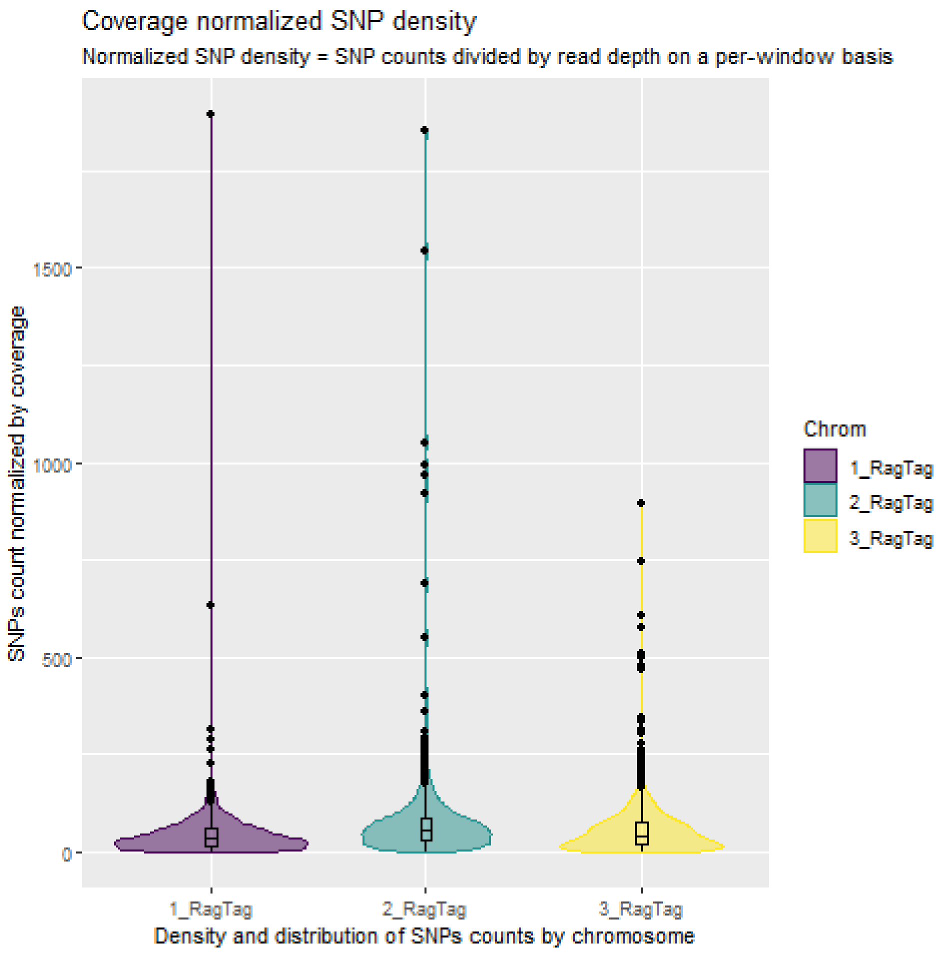

Again, boxes show interquartile range, whiskers show 1.5x interquartile range, with outliers shown as dots. This violin-wrapped boxplot shows the the distribution of coverage-normalized SNP density
on a non-transformed scale, which highlights the SNP counts of highly polymorphic regions. We lose resolution on the low end, however, because the distribution is so skewed.

Supplementary Data Figure S6 A log10 transformation of the y axis recovers low SNP count outlier windows.

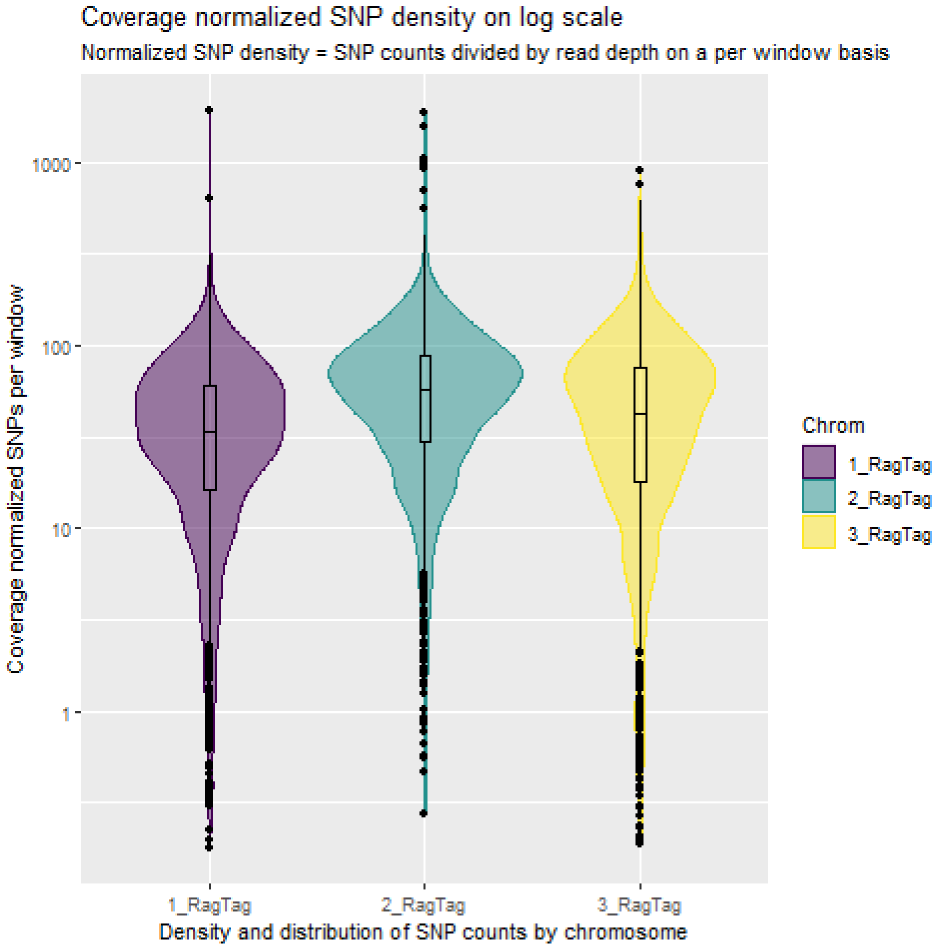

Normalized SNP density plotted against a log scale clarifies the occurrence of both high and low extreme outliers. Boxes show interquartile range, whiskers show 1.5x interquartile range, with outliers shown as dots.

Outliers here are pretty clear. The threshold for high SNP density is 500 or more SNPs per base mapped. The threshold for low SNP density is < 1 SNPs per base mapped. There are many more regions of low SNP density (100 windows) than high (15), which is interesting, and prompted us to investigate whether that was related to the density of genes in each region.

4) Gene density across the ROCK genome

In order to assess gene density, we used only the BRAKER gene models. Though we know these don't fully represent the genes that are likely present in the ROCK genome, they do represent nearly 80% of the genes annotated from VectorBase for AaegL5.3.

Supplementary Data Figure S7

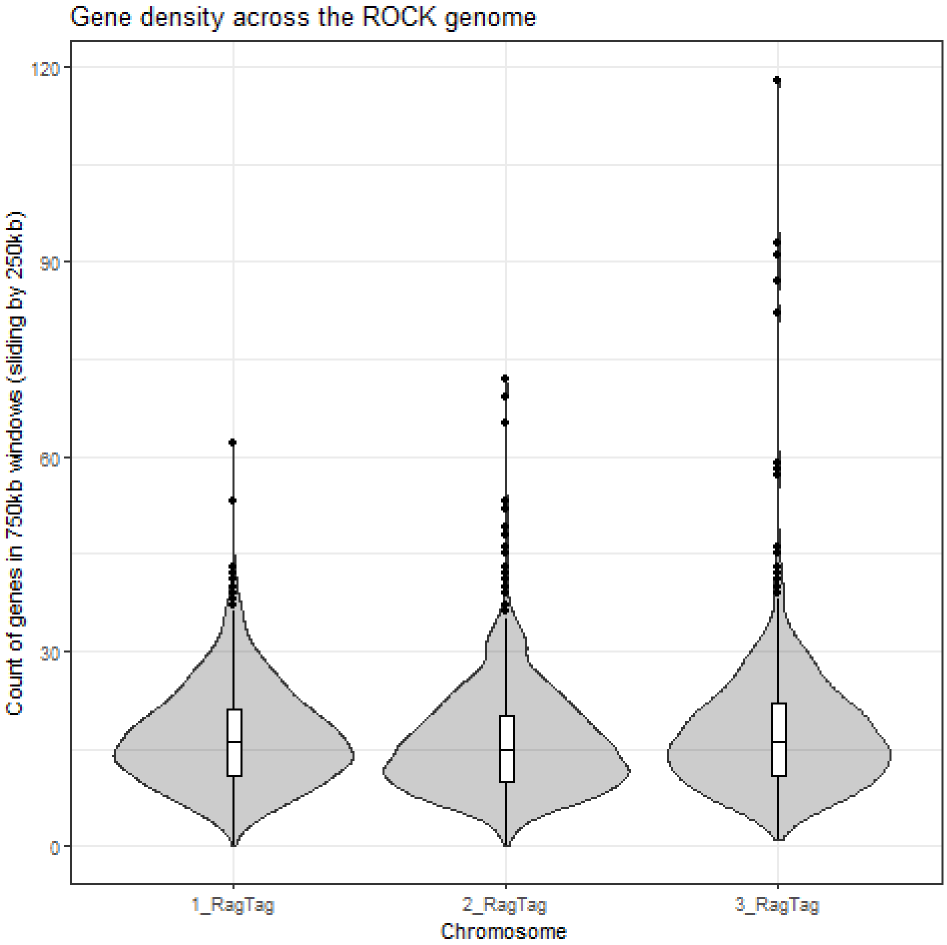

Mean gene density is about the same for each chromosome, and is 16.6 genes per 750kb window. The interquartile range is 11 to 21. There are 81 windows with gene count > 36 (3rd quartile + 1.5xIQR).

However, gene density and SNP density are uncorrelated (Pearson's R = -0.03), as is very clear from a basic scatterplot:

Supplementary Data Figure S8

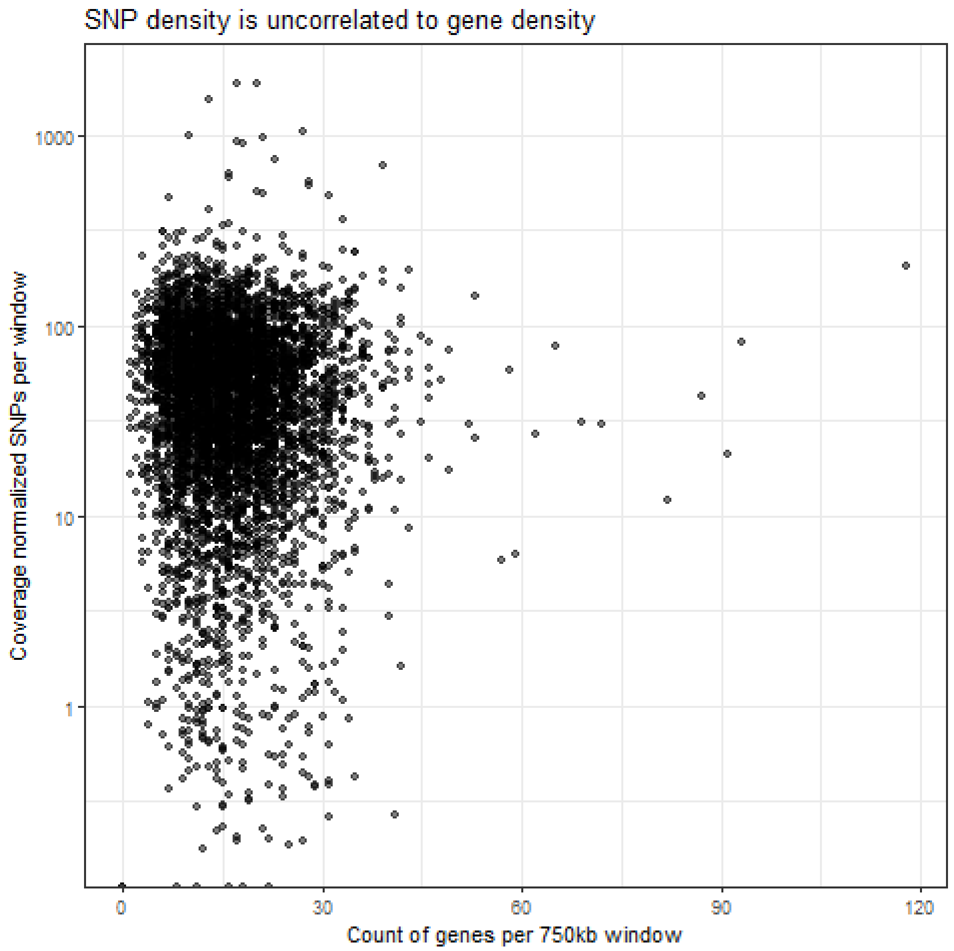

Supplement: jkac242_Supplementary_File_S2 [file jkac242_supplementary_file_s2.pdf]
