# Supplementary material for: A chromosome-level assembly of the widely used Rockefeller strain of Aedes aegypti, the yellow fever mosquito
Source: G3 (Bethesda). 2022 Sep 10;12(11):jkac242. doi: 10.1093/g3journal/jkac242 (PMC9635639; doi:10.1093/g3journal/jkac242)
Supplement: jkac242_Supplementary_Table_S1 [file jkac242_supplementary_table_s1.docx]

**Supplementary Table S1**

| RepeatMasker Outputs | | |  |  |  |  |
| --- | --- | --- | --- | --- | --- | --- |
|  |  |  | Model including "Unknowns" | | Model without "Unknowns" | |
|  |  |  | **ROCK** | **LVP5.3** | **ROCK** | **LVP5.3** |
| **Total bases masked** | | | 65.71% | 65.80% | 53.65% | 55.96% |
|  | Total interspersed repeats | | 59.76% | 59.95% | 47.78% | 49.82% |
|  |  | **Retroelements** | **19.86%** | **20.00%** | **19.77%** | **21.06%** |
|  |  | SINEs: | 1.67% | 1.69% | 1.85% | 1.89% |
|  |  | Penelope | 0.57% | 0.57% | 0.57% | 0.60% |
|  |  | LINEs: | 10.36% | 10.39% | 10.36% | 11.02% |
|  |  | CRE/SLACS | 0.00% | 0.00% | 0.00% | 0.00% |
|  |  | L2/CR1/Rex | 0.97% | 0.96% | 0.99% | 1.12% |
|  |  | R1/LOA/Jockey | 4.87% | 4.88% | 5.04% | 5.20% |
|  |  | R2/R4/NeSL | 0.05% | 0.05% | 0.05% | 0.05% |
|  |  | RTE/Bov-B | 1.29% | 1.28% | 1.17% | 1.31% |
|  |  | L1/CIN4 | 0.22% | 0.23% | 0.21% | 0.25% |
|  |  | LTR elements: | 7.83% | 7.93% | 7.56% | 8.15% |
|  |  | BEL/Pao | 1.06% | 1.11% | 0.87% | 1.12% |
|  |  | Ty1/Copia | 1.41% | 1.42% | 1.31% | 1.44% |
|  |  | Gypsy/DIRS1 | 3.98% | 4.00% | 4.00% | 4.16% |
|  |  | Retroviral | 0.00% | 0.00% | 0.00% | 0.00% |
|  |  | **DNA transposons** | **27.26%** | **27.23%** | **27.75** | **28.49%** |
|  |  | hobo-Activator | 1.68% | 1.64% | 1.72% | 1.78% |
|  |  | Tc1-IS630-Pogo | 0.39% | 0.39% | 0.37% | 0.40% |
|  |  | En-Spm | 0.00% | 0.00% | 0.00% | 0.00% |
|  |  | MuDR-IS905 | 0.00% | 0.00% | 0.00% | 0.00% |
|  |  | PiggyBac | 0.03% | 0.03% | 0.02% | 0.03% |
|  |  | Tourist/Harbinger | 0.48% | 0.47% | 0.47% | 0.48% |
|  |  | Other (Mirage, P-element, Transib) | 0.94% | 0.95% | 0.96% | 0.99% |
|  |  | Rolling-circles | 0.01% | 0.01% | 0.00% | 0.01% |
|  |  | **Unclassified:** | **12.65%** | **12.71%** | **0.26%** | **0.27%** |
|  |  |  |  |  |  |  |
|  |  | Small RNA: | 1.67% | 1.69% | 1.85% | 1.89% |
|  |  | Satellites: | 5.20% | 5.13% | 5.01% | 5.33% |
|  |  | Simple repeats: | 0.70% | 0.67% | 0.79% | 0.74% |
|  |  | Low complexity: | 0.05% | 0.04% | 0.07% | 0.06% |
